# A Local Genetic Algorithm for the Identification of Condition-Specific MicroRNA-Gene Modules

**DOI:** 10.1155/2013/197406

**Published:** 2013-01-21

**Authors:** Wenbo Mu, Damian Roqueiro, Yang Dai

**Affiliations:** Department of Bioengineering, University of Illinois at Chicago, Chicago, IL 60607, USA

## Abstract

Transcription factor and microRNA are two types of key regulators of gene expression. Their regulatory mechanisms are highly complex. In this study, we propose a computational method to predict condition-specific regulatory modules that consist of microRNAs, transcription factors, and their commonly regulated genes. We used matched global expression profiles of mRNAs and microRNAs together with the predicted targets of transcription factors and microRNAs to construct an underlying regulatory network. Our method searches for highly scored modules from the network based on a two-step heuristic method that combines genetic and local search algorithms. Using two matched expression datasets, we demonstrate that our method can identify highly scored modules with statistical significance and biological relevance. The identified regulatory modules may provide useful insights on the mechanisms of transcription factors and microRNAs.

## 1. Introduction

Transcription factors (TFs) and microRNAs exert a widespread impact on gene expression. Most genes in genome are regulated by the TFs, which account for about 10% of the protein-coding genes in humans and mice [1]. TFs function by interacting with genomic cis-regulatory DNA elements. MicroRNAs primarily bind to regulatory elements located in the 3′ untranslated region (3′UTR) of their target mRNAs. There are more than 1000 microRNAs, which target 60% of protein-encoding genes in the human genome, and each microRNA regulates about 200 transcripts (miRBase 2011 [2]). The identification of TF and microRNA targets is a key in understanding their roles in gene regulation. However, it is a laborious task. The availability of large amount of matched condition-specific microRNA and mRNA expression data for a specific cell or tissue type has provided a good resource for the prediction of microRNA functional target. Various methods using matched expression profiles coupled with sequence-based predictions of targets of microRNAs have been proposed [3]. On the other hand, the interplay between TFs and microRNAs was recently recognized [4]. However, there are only a limited number of integrated analysis tools [5–7]. Integrated analysis tools for identifying functional regulatory modules involving microRNAs and TFs targets are still needed.

## 2. Materials and Methods

**Figure 1:**
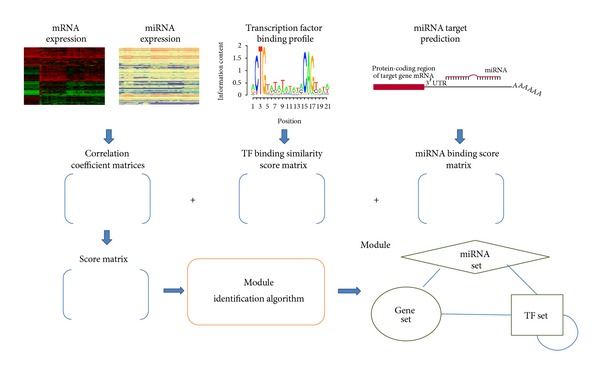
Method scheme.

The proposed method starts with a matched global mRNA and microRNA expression dataset; that is, mRNA and microRNA expression levels were measured from the same sample. The method consists of four steps. (1) Perform differential expression analyses for microRNA and mRNA profiles. (2) Calculate correlations of expression for pairs of microRNAs, pairs of mRNAs, and pairs of microRNAs and mRNAs. (3) Predict TF and microRNA targets. (4) Predict microRNA-gene modules based on the information obtained from (1) to (3) by a heuristic method, which is the combination of a genetic algorithm and a local search. The framework of our proposed method is presented in Figure 1.

### 2.1. Datasets and Preprocessing

Two datasets were used in our study. The first dataset contains the expression profiles of 98 primary cancer, 13 metastatic cancer, and 28 normal prostate samples [8]. The mRNA expression profiles were measured using the Affymetrix Human Exon 1.0 ST Array which includes 26,447 mRNAs, and the microRNA expression profiles were measured by the Agilent Human microRNA Microarray 2.0 which includes 368 microRNAs. The normalized data were obtained from the NCBI Gene Expression Omnibus (GEO) [9] through GEO accession number GSE21032. The second dataset includes a wide variety of tumor and normal tissue types: 218 tumor samples of 14 common tumor types and 90 normal tissue samples [10]. The mRNA expression profiles were measured with the Affymetrix Hu6800 and the Hu35KsubA Genechips and contained 16,063 genes. The corresponding microRNA expression profiles were measured with the bead-based flow cytometric microRNA expression profiling method on 217 mammalian microRNAs and 334 samples [11]. Among them, 68 cancer tissue samples on 11 tumor types and 21 normal samples have both mRNA expression profile and microRNA expression profile. These matched profiles were selected in our study. The normalized and log2-transformed data were obtained from the Broad Institute website (http://www.broad.mit.edu/cancer/pub/migcm/). 

The differential expression analysis was performed on both mRNA and microRNA expression profiles. Prior to the analysis, 25% probes with the lowest variation (measured by coefficient of variation) for both mRNAs and microRNAs were discarded. The differential expression analysis was performed using *limma* package in Bioconductor, and the false discovery rate (FDR) was controlled by adjusting *P* values based on the Benjamini and Hochberg multiple testing procedure [12]. Since functional TFs are not necessarily differentially expressed, all genes whose protein products are TFs (TF genes) were kept in our analysis. For the rest of the genes (nTF genes), a stringent cutoff of 0.001 for the adjusted *P* values was applied. Since a slight change of microRNA expresses can affect gene expression drastically, microRNAs with the adjusted *P* values less than 0.05 were defined as differentially expressed. 

Pearson correlation coefficients (PCCs) were used to measure correlations of expression of (1) mRNA pairs, (2) microRNA pairs, and (3) mRNA-microRNA pairs. A permutation test on PCCs was employed for significance analysis. Specifically, random expression profiles were generated by shuffling the mRNA labels in the original datasets for 10,000 times, and the PCC was recalculated for each shuffled dataset. The *P* value was determined as the percentage of times that the PCC obtained from a shuffled dataset exceeded that obtained from the observed data. 

Predicted microRNA targets were retrieved from the http://www.microRNA.org/ website, which provides access to the comprehensive database of predicted and experimentally validated microRNA targets [13–15]. The predicted targets for the conserved microRNAs with *P* value less than 0.05 were selected, resulting in a final set of 879,049 microRNA-gene pairs. The corresponding alignment scores associated with the microRNA targets were scaled to (0,1).

The predicted transcription factor binding sites (TFBSs) were obtained by mapping position weight matrices (PWMs) from TRANSFAC (ver. 2010.1) [16] of transcription factors to the promoter regions of genes using the MATCH algorithm [17]. We defined 10 KB upstream and 2 KB downstream of the transcription start site (TSS) as the promoter region of a gene. TFBSs were obtained from bindSDb [18], a database developed to store both experimentally validated and predicted TFBSs based on the RefSeq gene information from the UCSC RefSeq track of the Human Genome Assembly (hg19) and the NCBI mRNA annotations. In case there are multiple PWMs for a TF, the maximum alignment score of all its PWMs to the predicted TFBSs was used to determine the unique relation between the TF and its multiple PWMs. The matching information between a TF and its gene symbol was obtained from TRANSFAC. Even with the stringent threshold for the alignment scores, the MATCH algorithm still produced a large number of TFBSs, among which many may be false positives. To reduce the number of false positives, we applied a cutoff value (described later) on the similarity scores to reduce the number of interactions significantly without losing too much information.

### 2.2. Proposed Algorithm

Our module identification method consists of two steps. (1) Identify coexpressed gene sets which include TF genes and nTF genes by the genetic algorithm (GA). This step located the highly plausible region of “good” solution in the searching space. (2) Search coregulators for the coexpressed gene sets obtained by the GA using the local search algorithm. All direct regulators of genes were candidates for the local search. In order to guarantee no duplicated modules to be considered in the future generations, after a module was identified from the local search, the correlation coefficient matrix of mRNAs was updated by removing the pairs involving the mRNAs in the current module. The pseudocode of our algorithm is given in Algorithm 1.

#### 2.2.1. Design of the Genetic Algorithm

A binary string of fixed length was used to represent a chromosome, that is, a candidate of coexpressed gene sets in the GA. The value 1 stands for the gene included in the set and 0 for otherwise. Three setups with different percentages of genes included in the initial chromosomes were considered: 2%, 20%, and 80% of total genes. The roulette wheel selection was used for the selection of parent chromosomes for producing offspring. For the selected parents, the crossover was carried out separately for TF genes and nTF genes. The crossover probability *P*_co⁡_ was in the range of (0.5–0.9) with an incremental size of 0.1. The mutation probability *P*_mu_ was varied at four values: 0.00001, 0.0001, 0.001, and 0.01. In addition to these genetic operators, randomly generated chromosomes were introduced as new immigrants into the population pool to substitute the worst chromosome at each generation. Three immigration rates, 0.01, 0.001, and 0.0001, were considered. 

The average of the absolute PCCs over all pairs of genes included in a chromosome was defined as the fitness score of the chromosome. Two termination conditions were considered: 5,000 generations limitation or the highest fitness score remains unchanged for 200 generations. 

#### 2.2.2. Design of Local Search Algorithm

After the best coexpressed gene set was obtained from the GA, the candidates for the local search were determined to be all regulators (microRNAs and TFs) that were either predicted to target the genes in the coexpressed gene set or had significant PCCs with them. The initial solution for the local search was constructed by the TF genes in coexpressed genes and the randomly added 1% microRNAs from the candidate pool of regulators. The fitness score of a local search solution, or module, was defined as follows. 

Let *M*′ and *T*′ represent the set of microRNAs and TF genes in the module, respectively, *G*′ the union of both TF genes and nTF genes, *N* the total number of interactions among the members in the module. 

Define MGI as a score for the predicted targeting interactions between microRNAs and genes; MS_*ij*_ and Cor_*ij*_ as the binding score and the correlation coefficient between microRNA *i* and gene *j*, respectively:

(1) $$\begin{array}{c} \text{MGI}=\sum_{i\in M^{\prime }}\sum_{j\in G^{\prime }}\left(k_{1}{\text{MS}}_{ij}+k_{2}\left|{\text{Cor}}_{ij}\right|\right). \end{array}$$

Here *k*_1_ and *k*_2_ are two parameters. In our study we used *k*_2_ = 1 and *k*_1_ = 1,2, 3. 

Define TGI as a score for the predicted target interactions between TF genes and all genes; TS_*ij*_ and Cor_*ij*_ as the binding score and correlation coefficient between TF-gene *i* and nTF-gene *j*, respectively:

(2) $$\begin{array}{c} \text{TGI}=\sum_{i\in T^{\prime }}\sum_{j\in G^{\prime }}\left({k_{1}\text{TS}}_{ij}+k_{2}\left|{\text{Cor}}_{ij}\right|\right). \end{array}$$

The total PCCs among microRNAs in *M*′ were denoted by Cor_*M*′_:

(3) $$\begin{array}{c} {\text{Cor}}_{M\prime }=\sum_{i,j\in M^{\prime }}\sum_{i\neq j}\left|{\text{Cor}}_{ij}\right|. \end{array}$$

To prevent the size of modules from unlimited increasing, the fitness score for a module was defined as the averaged value over the four sets of interaction scores described above:

(4) $$\begin{array}{c} F=\frac{\text{MGI}+\text{TGI}+{\text{Cor}}_{M^{\prime }}}{N}. \end{array}$$

The interaction scores of TF-gene and microRNA-gene and all absolute PCCs were further scaled in the range of (0.5–1). The local search was terminated either when it reached 1000 iterations or the fitness scores remained unchanged for 100 iterations.

At each iteration of the local search, a local change to either microRNAs or TFs was made. For the user's convenience, we added a user option that specifies a preferred size of regulators in local search, since in most circumstances a user may be only interested in several most important regulators. For study reported here, the numbers of microRNAs and TFs in the modules are controlled at less than 1% and 4% of candidate regulators, respectively. After microRNAs/TF genes were determined to change, a microRNA/TF-gene was chosen from all candidates if the restriction of size had not been reached. A chosen microRNA/TF-gene was removed from the solution if it was already in the solution. If the number of the current regulators in the solution had reached the limit, a microRNA/TF-gene in the candidate searching space but not belonging to the current solution was chosen to substitute one microRNA/TF-gene in the current solution. 

### 2.3. Validation and Evaluation Criteria

In order to evaluate the overall quality of the identified modules, we defined a score by combining the fitness measurements used in the GA and local search. In addition to the fitness measurement used in local search, a term of total correlation coefficients among nTF genes in the module, Cor_*R*′_, was added:

(5) $$\begin{array}{c} {\text{Cor}}_{R^{\prime }}=\sum_{i,j\in R\prime }\sum_{i\neq j}\left|{\text{Cor}}_{ij}\right|. \end{array}$$

The final score for an identified module was defined as below:

(6) $$\begin{array}{c} F=\frac{\text{MGI}+\text{TGI}+{\text{Cor}}_{M^{\prime }}+{\text{Cor}}_{R^{\prime }}}{N}, \end{array}$$

where *N* is the total number of interactions among the members in the module. 

In order to show our method can successfully identify modules with high fitness scores, we compared specific scores of randomly generated modules with the identified modules. For each module, 1,000 randomized controls were generated and each control has the identical number of microRNAs, TF genes, and nTF genes with the identified modules. To evaluate the significance of our modules, we performed the permutation test for each module to determine *P* values. For each module at each permutation, a number of microRNAs/genes in module were substituted by the same number of randomly selected microRNAs/genes. The size of substitutions follows a discrete uniform distribution between 0 and the number of genes for each identified module. The *P* value was evaluated by the chance of obtaining a permutated module better than the original one. To evaluate the biological relevance of our modules, we performed the enrichment analyses for gene ontology (GO) terms and KEGG pathways for the identified modules using DAVID [19]. 

## 3. Results and Discussion

In this section, we first show how to determine the parameter values in our algorithm using Dataset I. Subsequently, we present the predicted modules based on the determined parameters for Dataset I. Most of the results were derived based on *k*_1_ = *k*_2_ = 1 unless otherwise is specified.

We identified 1,933 differentially expressed nTF genes and 144 differentially expressed microRNAs for Dataset I. These 1,933 nTF genes, 189 TF genes with mRNA measurements, and 144 microRNAs were used to calculate PCCs of their expression levels. Only those PCCs with *P* value less than 0.0001 were considered to be significant and were retained for the subsequence analysis (See Table S1 in Supplementary Material available online at http://dx.doi.org/10.1155/2013/197406.)

To determine the cutoff value on the TFBS similarity scores, we checked the effect of different thresholds on the predicted number of TF-gene pairs. A total of 16,292,671 alignments between PWMs and TFBSs were obtained from bindSDb based on the TRANSFAC threshold for the minimum false positives, and 3,469,371 TF-gene pairs were specified after determining the unique TFBS for a TF as described in Section 2. The different numbers of predicted TF-gene pairs and the numbers of involved TFs based on different thresholds for similarity scores were summarized in Table S2. We applied a cutoff 0.99 for the similarity scores, which significantly reduced the number of predicted pairs without drastically changing the numbers of TFs and target genes. Finally 1,705,837 predicted pairs between 260 TFs and 21,054 genes were retained for the module identification. 

### 3.1. Determination of GA Parameters

We examined the average sizes of coexpressed gene sets obtained from the GA at three different sizes for the initial chromosomes setups, that is, inclusion of 2%, 20%, and 80% of genes. The average sizes of the coexpressed gene sets obtained from the GA were 54, 224, and 401, respectively. However, in the latter two cases, the fitness scores are far from converging at the termination. Therefore, we set the initial chromosomes with only 2% of randomly selected genes.

The proper choice of values for *P*_co⁡_, *P*_mu_, and *P*_new_ is important to the performance of a GA. To find the good value for each genetic operator, we ran the GA by changing the value of one operator while keeping the other two fixed. For each value of a specific operator, we ran genetic algorithm for 10 times, 1000 generations each, and evaluated the performance by convergence rate. The convergence rate was defined as average incensement of fitness score per iteration. The GA performed better with *P*_co⁡_ = 0.7, *P*_mu_ = 0.001, and *P*_new_ = 0.01 (Table S3). We used these values for the subsequent analysis.

### 3.2. Evaluation of Local Search

In order to demonstrate that the local search can find a local optimal solution, we recorded the start and end scores and calculated the convergence rate of the scores. The results for 10 modules (Figure S1) show that the local search did improve the fitness score and locate the local optimal solutions efficiently. 

### 3.3. Module Evaluation

**Figure 2:**
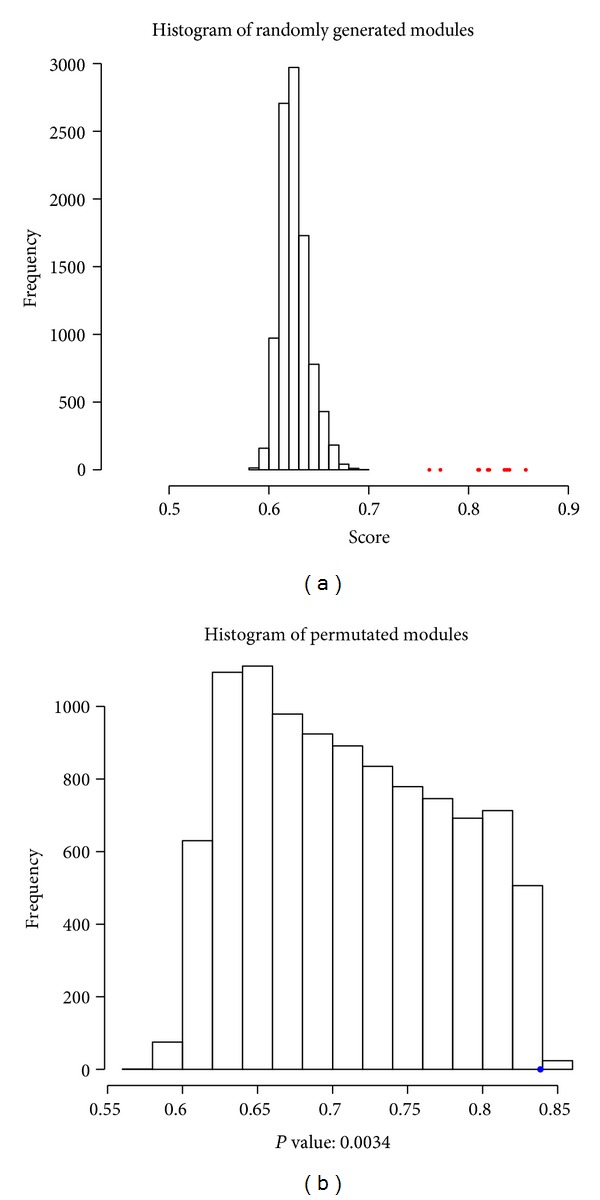
Histogram of control scores: (a) randomly generated modules; (b) permutated modules for module 1.

Figure 2(a) shows the histogram of fitness scores for 10,000 randomized modules and modules identified by our method (red dots). It suggests that our method was able to successfully identify modules with significantly higher scores. The identified modules, the corresponding scores, and the *P* values were listed in Table S4(a) (Supplementary file). All the modules were significant with *P* values less than 0.005 based on the permutation test.  Figure 2(b) shows the distribution of scores for the 10,000 permuted modules of module 1. It indicates that the local optimal solution was found by our method.

Table 1 provides a summary of the 10 regulatory modules found by our method. The interactions were divided into three categories based on the evidence of support: sequence-based binding prediction only, PCC only, and both. Most interactions predicted by sequence information also have significant PCCs, indicating the direct regulations. However, considerable fractions of interactions in the modules only have PCC support, implying indirect regulation between the regulators and targets. 

The details of genes and microRNAs in the identified modules, enriched KEGG pathways and GO terms (adjusted *P* < 0.01) were included in Tables S4(b) and S4(c) (Supplementary File). The enriched GO terms that annotate at least 5 genes were summarized. Compared to the results of enrichment analysis for the modules identified with a lasso model for the same dataset [20], most of the common KEGG pathways related to cancers were found, including focal adhesion, MAPK signaling pathway, hypertrophic cardiomyopathy, vascular smooth muscle contraction, regulation of actin cytoskeleton, pathways in cancer, and Wnt signaling pathway. 

### 3.4. Control of Interaction Types in the Predicted Modules

**Figure 3:**
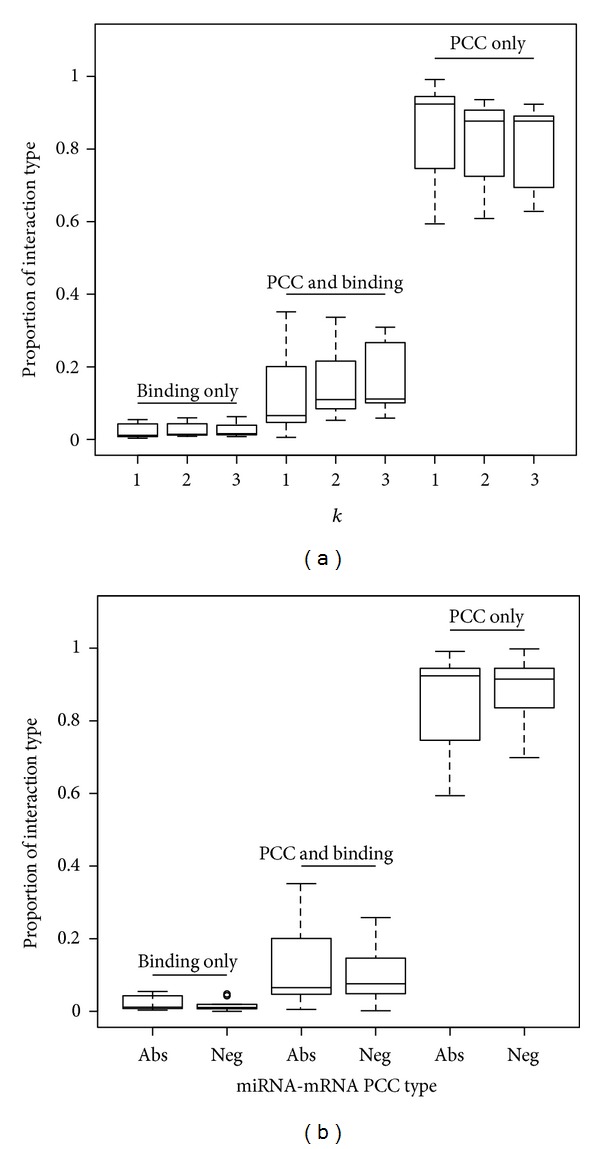
Boxplots of the proportion of three interaction types in the identified 10 modules with definitions for the fitness score. (a) The three boxplots in each type represent the results for (*k*_1_ = 1, *k*_2_ = 1), (*k*_1_ = 2, *k*_2_ = 1), and (*k*_1_ = 3, *k*_2_ = 1), respectively. (b) The two boxplots in each type represent the results using (1) both positive and negative microRNA-mRNA PCCs and (2) negative microRNA-mRNA PCCs, respectively.

The definition of the fitness score is a key factor to control the type of interactions one wishes to include in the modules. In the previous section we reported the results when an equal weight, that is, *k*_1_ = *k*_2_ = 1, was imposed on the alignment scores of TFs/microRNAs and the correlation coefficients of expression in the fitness function. We examined if the increase of the weight on the alignment scores could lead to the increase of the number of interactions with support from both the predicted binding and significant PCC values. We performed the experiment using *k*_2_ = 2,3 and *k*_1_ = 1. It can be observed that an increasing trend in the proportion of interactions was supported by the predicted binding and expression correlation between the regulators and targets (Figure 3(a)) when *k*_2_ increases. This result shows the flexibility of our method in finding regulatory modules according to user's preference on the interaction types. 

We also examined the ability of our method in finding regulatory modules when only including microRNAs that were negatively correlated with the predicted genes in the coexpressed set in the local search step. Our algorithm was able to successfully identify significant modules (Supplementary File 1). Compared with the case where both negatively and positively expressed microRNA regulators were considered in a module, there was a slight increase in the proportion of the interaction type with support from both predicted binding and significant PCCs (Figure 3(b)). 

### 3.5. Literature Validation

**Figure 4:**
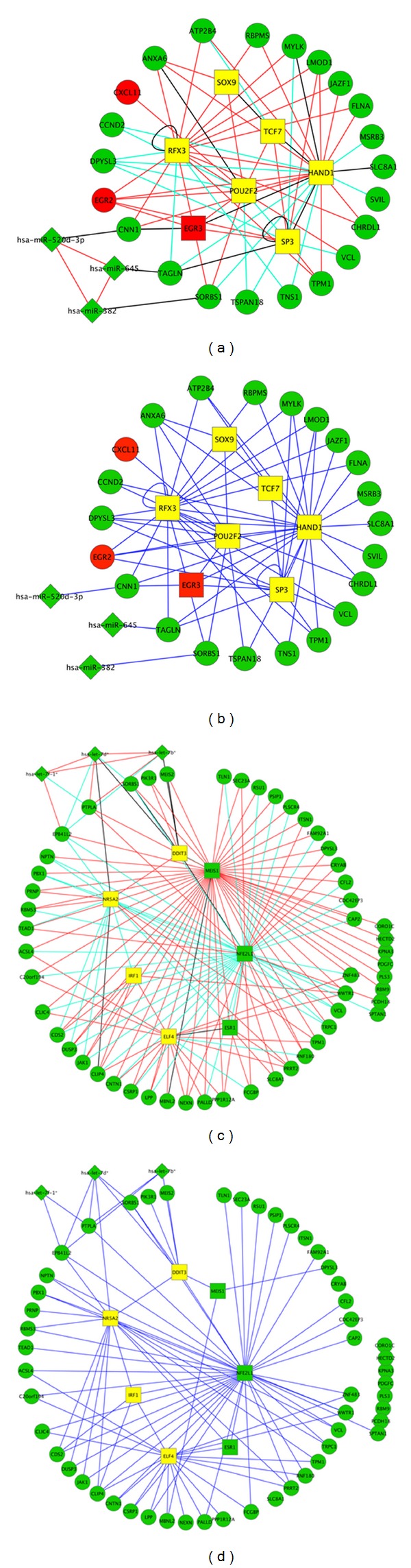
Two types of visualization of selected modules: (a) general representation of module 1; (b) sequence-based predictions only of module 1; (c) general representation of module 10; (d) sequence-based predictions only of module 10. Diamond, rectangle, and eclipse represent microRNA, TF genes, and nTF genes, respectively. Red nodes and green nodes represent overexpressed and underexpressed microRNAs/genes in tumor samples. Red lines and light green lines stand for positive correlations and negative correlations, respectively, while interactions that were predicted only by sequence information are drawn as black lines. For clear visualization, the links between nTF genes were not plotted.

The interactions in the identified module 1 to module 10 were shown in Figure 4 and Figures S2 and S3. In module 1, no microRNAs genes become isolated, and the main network structure is not changed after removing those predicted by the PCC interactions. In module 10, however, the targets of MEIS1 become isolated, and many potential regulatory relationships between MEIS1 and target genes also disappear after removing the PCC predicted interactions. MEIS1, which encodes a homeobox protein belonging to the TALE “three amino acid loop extension” family of homeodomain-containing proteins, as well as MEIS2 and PBX1 are found to have a critical function to suppress prostate cancer initiation and progression [21]. The difference between Figures 4(c) and 4(d) suggests that MEIS1 may be a coactivator to regulate genes without directly binding to the promoters of the targets.

We also explored the literatures about other core regulators and regulatory relationships in identified modules. For example, hsa-miR-7f-1, which was identified as a core regulator in both modules 8 and 10, was found to be associated with lung cancer, breast cancer, colorectal cancer [22], pancreatic cancer [23], and pituitary adenomas [24]. Another microRNA, hsa-miR-328, identified in modules 1, 3, 7, and 9, was found to be dysregulated in both breast cancer [25] and colorectal cancer [26]. But their functional mechanisms to cancer development are still unknown. Our predicted modules may be used to facilitate further experiments for functional study. 

Our method also identified several important TFs and their regulatory relationships, such as EGR3, RFX3 and MYLK. EGR3 was found overexpressed in tumor cells and was identified as a core regulator in modules 1, 3, and 10. The protein encoded by EGR3 participates in the transcriptional regulation of genes in controlling biological rhythm and may also play a role in a wide variety of processes including muscle development, lymphocyte development, endothelial cell growth, and migration and neuronal development (Ref-Seq December 2010). EGR3 was found to be closely associated with the genesis and malignant progression of breast cancer by being involved in the estrogen-signaling pathway. Recently it was shown that EGR3 plays a pivot role in mechanism of prostate cancer initiation or early progression [27]. RFX3 is a transcriptional activator with highly conserved winged helix DNA-binding domain and can bind DNA as a monomer or as a heterodimer with other RFX family members. Its function in prostate cancer has not been well explored, but its regulation on the same set of genes together with MEIS1 in modules 7 and 9 suggests it may present as a coregulator with MEIS1 to be functional. MYLK was known to be involved in many biological processes including the inflammatory response (e.g., apoptosis, vascular permeability, and leukocyte diapedesis), cell motility and morphology and MARK signaling pathway. It was identified to be coregulated by MEIS1 and RFX3 in modules 7 and 9. It was involved in a total of 5 modules, suggesting its importance in cancer development, especially prostate cancer. A thorough literature search on all of the predicted interactions and core regulators for prostate cancer is not possible here. However, we demonstrated that our method is likely to be useful for identifying functional regulatory modules in specific diseases. 

### 3.6. Prediction in Dataset II

Since Dataset II includes expression on a wide variety of tissue and normal samples, we applied our method to identify cancer-related common regulatory modules. Because of the multiple cell types and intrinsic complication of tumor cellular environment, we used the same procedure for differential expression analysis with a relatively loose cutoff for *P* values. The threshold of 0.05 for the adjusted *P* values was applied to the nTF genes and microRNAs. All TF genes were retained. This step resulted in 94 microRNAs, 162 TF genes, and 1,410 nTF genes. The sequence-based prediction for TF and microRNA targets led to a set of 74 microRNAs, 148 TF genes, and 1,194 nTF genes for module identification. We performed the same test to determine the optimal values for genetic operators. Crossover probability 0.7, mutation probability 0.001, and random immigrant probability 0.01 were obtained. These values were the same as those used for Dataset I, showing that the choice of parameters was not biased to a particular dataset. 

All 10 modules achieved the significance level based on our permutation test. The numbers of interactions in the identified modules are showed in Table S5. All sequence-base predicted regulations were with significant PCC values between the regulators and regulated genes. The enriched GO terms and KEGG pathways include cancer relevant GO terms and KEGG pathways, such as positive regulation of RNA metabolic process and Wnt signaling pathway, suggesting the method also predicted microRNA-gene regulatory modules for Dataset II (Table S6(b), Supplementary File).

## 4. Discussion

Several related methods and databases for the identification of microRNA-TF-gene regulatory modules have been published. The method we proposed has a number of advantages over other module identification methods. For example, CircuitDB [7] and MIR@NT@N [5] utilized sequenced-based target predictions and protein-protein interactions to constrict microRNA-TF-gene module. But they are static databases and could not answer the question about alteration of gene expressions in a specific type of disease or lack of ability to incorporate the expression values into analysis. MAGIA2 [28] and miRGator 2.0 [29] provided tools for prediction of microRNA-gene modules by combining the sequence-based target prediction and user-supplied expression profiles, but they did not separate TF genes from the entire set of genes. In regulatory modules, many TF genes that are not differentially expressed could be as important as differentially expressed TF genes as coactivators. mirConnX [6] took both the sequence-based predictions and the specified TFs into consideration to construct condition-specific mRNA-microRNA networks. However, the resulting networks were often too large to pinpoint the most important functional modules in a disease. Our method bridged the gap between the above methods by utilizing both sequence-based predictions and expression profiles and emphasizing the transcription factor's effect for the detection of condition-specific regulatory modules.

Our method and other similar methods that identify microRNA-gene regulatory modules were based on the assumption that microRNAs are posttranscriptional regulators that regulate TFs' expressions. But several studies have proposed that TFs can regulate the transcription of microRNA directly [30, 31]. Currently those databases were built to predict the regulation of transcription factors on microRNA precursors. A possible extension of our method is to transfer it into relationships between transcription factors and mature microRNAs and to incorporate this knowledge into our module identification method.

As more information is incorporated, not all of them should be considered equally in evaluation; for example, experimentally validated regulation may be more valuable for the user. In addition, the microRNA's regulation on TF genes and nTF genes are measured equally in current method, but the microRNA's regulation on TF genes may be more interesting. We can improve our method by adding control parameters to emphasize specific types of relationships.

## 5. Conclusion

We proposed a computational method that combines the sequence-based target predictions and matched microRNA-gene expression profiles. Our method independently processes measurement of interactions, identification of coexpression gene sets, and regulatory modules. The major characteristics are (1) easy integration of other methods for identification of gene coexpression set, (2) easy refinement by including updated information of target prediction, and (3) easy setup of parameters to emphasize interest of research. It is a candidate tool for clinical researchers to use user-supplied data to perform further investigation and exploration.

## Supplementary Material

The supplemental file presents the supporting material on the determination of the thresholds for various p-values used in the algorithm. Additional analysis results for Dataset I and Dataset II based on the proposed algorithm are also included.

## Figures and Tables

**Algorithm 1:**
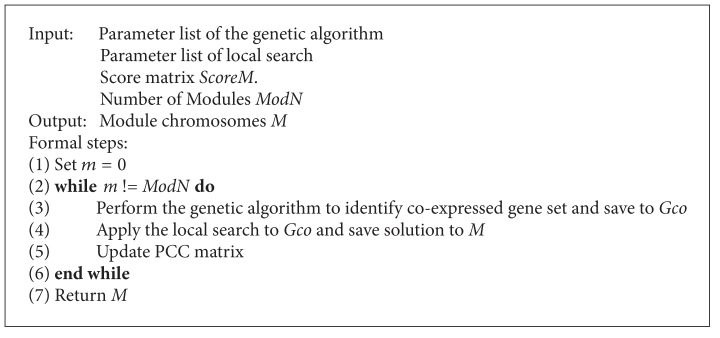
Pseudocode for module identification.

**Table 1:** Summary of regulatory interactions in the 10 predicted modules for Dataset I.

Module ID	# Nodes^a^	# Interactions^b^	# PCC and Binding^c^	# PCC^d^	# Binding^e^
1	3/7/22	264	53	197	14
2	3/3/36	704	33	665	6
3	3/7/39	823	60	751	12
4	3/15/21	384	135	228	21
5	3/7/17	233	74	149	10
6	3/3/49	1284	7	1273	4
7	3/4/46	1181	42	1127	12
8	3/7/42	988	99	877	12
9	3/7/49	1316	74	1232	10
10	3/7/53	1431	83	1339	9

^a^The numbers of miRNAs, TF-genes, and nTF-genes.

^b^The number of interactions.

^c^The number of interactions with support of both significant PCC and predicted binding.

^d^The number of interactions with support of only significant PCC.

^e^The number of interactions with support of only predicted binding.
