# Supplementary material for: A Local Genetic Algorithm for the Identification of Condition-Specific MicroRNA-Gene Modules
Source: ScientificWorldJournal. 2013 Jan 21;2013:197406. doi: 10.1155/2013/197406 (PMC3564382; doi:10.1155/2013/197406)
Supplement: Supplementary file 1 — The supplemental file presents the supporting material on the determination of the thresholds for various p-values used in the algorithm. Additional analysis results for Dataset I and Dataset II based on the proposed algorithm are also included. [file 197406.f1.pdf]

Supplementary file

Biological relevance of the modules identified for Dataset II

Visualization of identified modules is showed in Figures S4-S5. Many core regulators and regulatory relationships have been verified by other literatures. For example, has-mR-15a that appeared in two identified modules was identified as regulators in multiple cancer types. It was shown that has-miR-15a was down-regulated in the majority of non–small cell lung cancer to induce cell cycle arrest in G1-G0 in a Rb-dependent manner [1]. A study on kidney cancer also suggested has-miR-15a may be involved in cancer pathogenesis and has the potential to be biomarkers [2]. Another research proposed that miR-15a acted as tumor suppressor genes in prostate cancer through the control of cell survival, proliferation and invasion [3]. A research using breast cancer samples validated this conclusion by showing that has-miR-15a can suppress cancer stem cells growth and hypothesizing it may play a pivotal role in multiple cancers [4]. Bhattacharya revealed possible development of therapeutic strategies by restoring has-miR-15a expression in ovarian cancer and in other cancers [5]. Similarly, has-miR-19a was identified as a core regulator in 4 identified modules, its expression was also found significantly associated with colorectal cancer [6], lung cancer [7] and multiple myeloma [8]. Another study demonstrated hsa-miR-19b as co-regulator with has-miR-19a in regulating the IL-6 antiapoptotic signaling in the pathogenesis and malignant growth of multiple myeloma [8].

Besides core microRNAs, core transcription factors in our identified modules are verified to be relevant with multiple cancers as well. For example, ARNT2, aryl hydrocarbon receptor nuclear translocator, was annotated as a pivot gene in cancer related pathways by KEGG. And many studies have showed that its differential expression maybe associated with disease outcome of colon cancer [9] and lung carcinoma [10]. However its regulatory mechanism has not been well explored by available researches. Its involvement in three identified modules may provide guidance for further experimental design. SMAD3, an intracellular signal transducer and transcriptional modulator, was found overexpressed in advanced human prostate cancer and critical for progression of prostate cancer cells [11]. And our identified modules suggest it may also promote the progressive growth of other type of tumor cells. TP53 (p53) is a well-explored gene that regulates a large number of genes and thus controls a number of key tumor suppressing functions such as cell cycle arrest, DNA repair, senescence and apoptosis. TP53 was identified as core transcription factor regulators in module 2, 7, and 10. Even it was not differentially expressed based on stringent p value cutoff, its positive correlation with the regulated genes suggested that its inactivation may lead to tumor progression. Similarly, MYB is another famous oncogene that plays an essential role in the regulation of hematopoiesis and may play a role in tumorigenesis [12]. Our regulatory module 3 suggests its responsibility for overall under-expression of regulated genes. GPR17, the overexpressed nTF-gene in module 4, is a Gi-coupled orphan receptor and its activation may lead to both adenylyl cyclase inhibition and intracellular calcium increase, which is also important in cell proliferation, differentiation, apoptosis, and cytotoxicity [13].

Supplementary Tables

Table S1: Summary of filtering steps for PCC by P-value cutoff

P value threshold	# of microRNA pairs / # of microRNAs	# of microRNA-mRNA pairs/ # of microRNAs/ # of mRNAs	# of mRNA-mRNA pairs/ # of mRNAs
Before Filtering	20,736 / 144	305,568 / 144 / 2122	4,502,884 / 2122
P value < 0.01	12,390 / 144	150,151 / 144 / 2122	3,298,516 / 2122
P value < 0.001	9,987 / 144	106,142 / 144 / 2122	2,838,129 / 2122
P value < 0.0001*	8,561 / 144	80,429 / 144 / 2078	2,495,043 / 2122

Table S2. Summary of filtering steps for TF predicted targeting by similarity score cutoff

Score Threshold	# of Interactions	# of TFs / # of Targets
Before filtering	3,469,371	265/21,054
> 0.85	3,359,558	265/21,054
> 0.90	3,133,653	265/21,054
> 0.95	2,631,890	262/21,054
> 0.99*	1,705,837	260/21,054
> 0.999	610,465	242/21,054

Table S3. Convergence rates for different GA parameters

Pco	Rates	Pmu	Rates	Pnew	Rates
0.5	2.27E-04	0.01	5.09E-05	0.01*	3.15E-04
0.6	2.45E-04	0.001	2.63E-04	0.001	2.71E-04
0.7*	3.84E-04	0.0001*	1.02E-03	0.0001	2.81E-04
0.8	2.69E-04	0.00001	4.97E-04		
0.9	2.61E-4				

Table S4 (A): Identified modules in dataset I

ID	microRNA	TF	nTF	Score	P value
1	hsa-miR-382<br>hsa-miR-520d-3p<br>hsa-miR-645	EGR3,HAND1,POU2F2,RFX3,SOX9,SP3,TCF7	ANXA6,ATP2B4,CCND2,CHRDL1,CNN1,CXCL11,DPYSL3,EGR2,FLNA,JAZF1,LMOD1,MSRB3,MYLK,RBPMS,SLC8A1,SORBS1,SVIL,TAGLN,TNS1,TPM1,TSPAN18,VCL	0.8386	3.40E-03
2	hsa-miR-382<br>hsa-miR-423-3p<br>hsa-miR-520d-3p	MEIS1,RFX3,SOX9	ACTA2,ATP2B4,CALD1,CAP2,CAV1,CCND2,CDC42EP3,CFL2,CHRDL1,CLU,CNN1,CORO1C,DDR2,DMD,DPYSL3,FHL1,FLNA,FRMD6,LMOD1,MAP1B,MSRB3,MYL9,MYLK,MYOCD,PALLD,PCP4,RBPMS,SEC23A,SLC8A1,SORBS1,SVIL,TIMP2,TLN1,TNS1,TPM1,VCL	0.8574	2.50E-03
3	hsa-miR-129-5p<br>hsa-miR-328<br>hsa-miR-645	EGR3,IRF1,POU3F1,RFX3,RUNX3,SOX9,TOPORS	ACTN1,AHNAK2,ANXA6,BNC2,CHRDL1,CLU,COL4A2,COL6A1,DES,DKK3,EDNRA,FLNA,FLNC,FZD7,GSN,HEPH,HTRA1,ITGA9,ITIH5,JAZF1,KCNMB1,LGALS1,LMOD1,MAP1B,MCAM,MFGE8,MRVI1,MYL9,MYLK,NID1,PMP22,RBPMS,SLC24A3,SLC8A1,TIMP2,TNS1,TPM2,TSPAN18,WFDC1	0.836	1.20E-03

Table S4 (A): Identified modules in dataset I

ID	microRNA	TF	nTF	Score	P value
4	hsa-miR-647<br>hsa-miR-877*<br>hsa-miR-885-5p	FOSL1,FOXA1,GLI1,HMGA2,JUND,MAZ,NKX2-5,PAX8,PDX1,RFX3,RUNX2,SOX9,STAT3,T,ZBTB7A	ASCC1,ATP6V1E1,CLASP1,CLCA2,DCTN2,DLST,DYNC1H1,FXYD6,GPX2,KIAA0430,MYST2,PARD3,PCDH10,PPP3CB,RBM23,SDHD,SERPINA11,SIGLEC12,TMEM132A,TOR1AIP1,ZFHX4	0.7608	2.20E-03
5	hsa-let-7d*<br>hsa-miR-548d-5p<br>hsa-miR-647	ETV4,FOSL1,GATA6,HAND1,RFX3,SOX9,STAT3	ACTN1,ANXA6,ATP2B4,CAV1,CCND2,CNN1,CSRP1,FLNA,GSN,ILK,SORBS1,SPTAN1,SVIL,TAGLN,TLN1,TNS1,TPM2	0.841	4.90E-03
6	hsa-miR-10b*<br>hsa-miR-625<br>hsa-miR-660	DDIT3,HLF,MEIS1	ACTG2,ACTN1,ANXA6,ATP2B4,CALD1,CAMK2G,CAV1,CCND2,CHRDL1,CLU,CNN1,CORO1C,CRYAB,CSRP1,DDR2,DES,DPYSL3,DUSP3,EDNRA,EYA4,FHL1,FLNA,FLNC,ILK,KCNMB1,LMOD1,MAOB,MATN2,MEIS2,MSRB3,MYLK,NEXN,NPTN,PALLD,PARVA,PPP1R3C,PRNP,RBPMS,SLC8A1,SVIL,TAGLN,TGFB2,TGFBR3,TIMP2,TLN1,TNS1,TPM1,TSPAN2,VCL	0.8194	3.10E-03

Table S4 (A): Identified modules in dataset I

ID	microRNA	TF	nTF	Score	P value
7	hsa-miR-328 hsa-miR-605 hsa-miR-645	MEIS1,PGR,RFX3,SREBF2	ACTN1,ADRA1A,ANXA6,ATP2B4,BNC2,CAV1,CHRDL1,CLU,CNN1,CNTN1,CORO1C,DDR2,DPYSL3,EDNRA,FAT4,FBN1,FLNA,GNAL,GSN,ITGB3,ITIH5,KCNMA1,LAMA4,LMOD1,MAP1B,MRVI1,MSRB3,MYH11,MYLK,MYOCD,PDZRN4,PMP22,RBMS2,RGN,SLC8A1,SORBS1,SSPN,SVIL,TIMP2,TMEM47,TNS1,TPM1,TPM2,TRPC1,TRPC4,VCL	0.8207	2.50E-03
8	hsa-let-7b* hsa-let-7d* hsa-let-7f-1*	BPTF,EGR3,HAND1,POU3F1,RFX3,SOX9,TGIF1	ADCY5,AHNAK2,CAV1,CHRDL1,CLU,CNN1,COL4A2,COL6A1,COL6A2,DBNDD2,DES,DKK3,DNAJB5,EFEMP2,ENO2,FGFR1,FHOD3,FLNC,FZD7,GAS6,GPR124,GSN,HEPH,ITGA7,ITGA9,ITGB3,ITIH5,JAZF1,LMOD1,MARVELD1,MCAM,MKX,MRGPRF,MRVI1,NFASC,PNMA1,PRICKLE2,RARRES2,SLC24A3,ST5,TSPAN18,WFDC1	0.8106	2.40E-03

Table S4 (A): Identified modules in dataset I

ID	microRNA	TF	nTF	Score	P value
9	hsa-let-7b*<br>hsa-let-7d*<br>hsa-miR-328	DDIT3,IRF1,JUND,MEIS1,RFX3,SOX9,ZBTB16	ABCC9,ANXA6,ATP2B4,C20orf194,CACHD1,CALD1,CAMK2G,CAP2,CAV1,CCND2,CFL2,CLIP4,CORO1C,DDR2,DMD,DPYSL3,EDNRA,EPB41L2,FRMD6,KLHL5,LTBP1,MAP1B,MFN2,MSRB3,NT5E,PALLD,PDGFC,PLS3,PRNP,PSIP1,RBMS2,RBMS3,RBPMS,SEC23A,SLC8A1,SORBS1,SPARCL1,SPTAN1,SVIL,TEAD1,TIMP2,TLN1,TNS1,TRPC1,TRPC4,TSPAN2,VCL,WWTR1,ZNF483	0.8097	1.90E-03
10	hsa-let-7b*<br>hsa-let-7d*<br>hsa-let-7f-1*	DDIT3,ELF4,ESR1,IRF1,MEIS1,NFE2L1,NR5A2	ACSL4,C20orf194,CAP2,CDC42EP3,CDS2,CFL2,CLIC4,CLIP4,CNTN1,CORO1C,CRYAB,CSRP1,DPYSL3,DUSP3,EPB41L2,FAM92A1,FCGBP,HECTD2,ITSN1,JAK1,KPNA3,LPP,MBNL2,MEIS2,NEXN,NPTN,PALLD,PBX1,PCDH18,PDGFC,PIK3R1,PLS3,PLSCR4,PPP1R12A,PRNP,PRRT2,PSIP1,PTPLA,RBM9,RBMS3,RNF180,RSU1,SEC23A,SLC8A1,SORBS1,SPTAN1,TEAD1,TLN1,TPM1,TRPC1,VCL,WWTR1,ZNF483	0.7718	3.50E-03

Table S4 (B): Enriched KEGG pathways of identified modules in dataset I

Module ID	KEGG pathways	P value
1	hsa04510:Focal adhesion	0.004283822
	hsa04520:Adherens junction	0.007606025
	hsa04020:Calcium signaling pathway	0.036540214
2	hsa04510:Focal adhesion	8.16E-06
	hsa04270:Vascular smooth muscle contraction	0.003171875
	hsa04810:Regulation of actin cytoskeleton	0.019206274
	hsa05410:Hypertrophic cardiomyopathy (HCM)	0.022059277
	hsa05414:Dilated cardiomyopathy	0.025583328
3	hsa04510:Focal adhesion	6.68E-07
	hsa04270:Vascular smooth muscle contraction	2.52E-04
	hsa05412:Arrhythmogenic right ventricular cardiomyopathy (ARVC)	0.001283014
	hsa05410:Hypertrophic cardiomyopathy (HCM)	0.001774001
	hsa05414:Dilated cardiomyopathy	0.002227637
	hsa04810:Regulation of actin cytoskeleton	0.002935721
	hsa04512:ECM-receptor interaction	0.024629357
4	NA	NA
5	hsa04510:Focal adhesion	5.80E-05
6	hsa04510:Focal adhesion	1.54E-07
	hsa04270:Vascular smooth muscle contraction	0.001686291
	hsa05410:Hypertrophic cardiomyopathy (HCM)	0.007080857
	hsa04020:Calcium signaling pathway	0.008566318
	hsa05414:Dilated cardiomyopathy	0.008810366
	hsa04010:MAPK signaling pathway	0.034423309
	hsa05412:Arrhythmogenic right ventricular cardiomyopathy (ARVC)	0.04922478
7	hsa04020:Calcium signaling pathway	5.56E-05
	hsa04270:Vascular smooth muscle contraction	7.28E-05
	hsa04510:Focal adhesion	1.17E-04
	hsa05410:Hypertrophic cardiomyopathy (HCM)	0.004823721
	hsa05414:Dilated cardiomyopathy	0.0060202
	hsa04810:Regulation of actin cytoskeleton	0.010549498
	hsa05412:Arrhythmogenic right ventricular cardiomyopathy (ARVC)	0.038540555
	hsa04520:Adherens junction	0.039470992
	hsa04260:Cardiac muscle contraction	0.040410096

Table S4 (B): Enriched KEGG pathways of identified modules in dataset I

Module ID	KEGG pathways	P value
8	hsa04510:Focal adhesion	1.15E-06
	hsa04512:ECM-receptor interaction	4.13E-06
	hsa05414:Dilated cardiomyopathy	1.55E-04
	hsa05412:Arrhythmogenic right ventricular cardiomyopathy (ARVC)	0.0015622
	hsa05410:Hypertrophic cardiomyopathy (HCM)	0.002157177
	hsa04810:Regulation of actin cytoskeleton	0.003786154
9	hsa04020:Calcium signaling pathway	0.006223127
	hsa04510:Focal adhesion	0.009899224
10	hsa04510:Focal adhesion	0.006973886
	hsa04810:Regulation of actin cytoskeleton	0.008826945

Table S4 (C): Enriched GO terms of identified modules in dataset I

ID	Term	Count	Fold Enrichment	Benjamini	FDR
1	GO:0003779~actin binding	9	13.275051	1.65E-05	1.72E-04
	GO:0008092~cytoskeletal protein binding	10	9.5407113	1.74E-05	3.63E-04
2	GO:0003779~actin binding	16	18.20578	1.13E-13	1.03E-12
	GO:0008092~cytoskeletal protein binding	18	13.247959	6.34E-14	1.15E-12
	GO:0005856~cytoskeleton	19	5.3289886	5.73E-08	4.61E-07
	GO:0015629~actin cytoskeleton	11	15.838909	4.58E-08	7.37E-07
	GO:0007010~cytoskeleton organization	12	10.950890	2.60E-06	5.33E-06
	GO:0043228~non-membrane-bounded organelle	21	3.1332819	1.17E-05	2.81E-04
	GO:0043232~intracellular non-membrane-bounded organelle	21	3.1332819	1.17E-05	2.81E-04
	GO:0030055~cell-substrate junction	6	20.75	2.91E-04	0.00937
	GO:0044449~contractile fiber part	6	20.566371	2.43E-04	0.00979
	GO:0043292~contractile fiber	6	19.206611	2.83E-04	0.01368
	GO:0006937~regulation of muscle contraction	5	27.630718	0.0099143	0.04080
	GO:0005924~cell-substrate adherens junction	5	18.270440	0.0028371	0.15997
	GO:0016323~basolateral plasma membrane	6	11.448275	0.0025243	0.16264
	GO:0005912~adherens junction	5	12.494623	0.0084641	0.68192
3	GO:0003779~actin binding	8	8.6108439	0.0035730	0.03089
	GO:0008092~cytoskeletal protein binding	9	6.2659266	0.0038333	0.06628
4	GO:0043565~sequence-specific DNA binding	13	8.4258898	1.02E-06	1.06E-05
	GO:0045944~positive regulation of transcription from RNA polymerase II promoter	10	11.394878	6.03E-05	1.40E-04
	GO:0003700~transcription factor activity	14	5.6491686	1.10E-05	2.27E-04
	GO:0006357~regulation of transcription from RNA polymerase II promoter	12	6.9779917	1.01E-04	4.70E-04
	GO:0045893~positive regulation of transcription, DNA-dependent	10	8.8626834	1.68E-04	0.00117
	GO:0051254~positive regulation of RNA metabolic process	10	8.7889812	1.35E-04	0.00125

Table S4 (C): Enriched GO terms of identified modules in dataset I

ID	Term	Count	Fold Enrichment	Benjamini	FDR
4	GO:0006355~regulation of transcription, DNA-dependent	16	3.8150028	2.66E-04	0.00309
	GO:0051252~regulation of RNA metabolic process	16	3.7308328	2.95E-04	0.00411
	GO:0006350~transcription	17	3.4206330	2.88E-04	0.00469
	GO:0045941~positive regulation of transcription	10	7.4955673	2.53E-04	0.00470
	GO:0010628~positive regulation of gene expression	10	7.2762478	2.87E-04	0.00600
	GO:0030528~transcription regulator activity	15	3.9030182	1.78E-04	0.00551
	GO:0045935~positive regulation of nucleobase, nucleoside, nucleotide and nucleic acid metabolic process	10	6.7748397	4.62E-04	0.01075
	GO:0051173~positive regulation of nitrogen compound metabolic process	10	6.5644409	5.42E-04	0.01387
	GO:0010557~positive regulation of macromolecule biosynthetic process	10	6.4640672	5.62E-04	0.01571
	GO:0045449~regulation of transcription	18	2.9256055	5.23E-04	0.01582
	GO:0031328~positive regulation of cellular biosynthetic process	10	6.1715328	7.00E-04	0.022806
	GO:0009891~positive regulation of biosynthetic process	10	6.0827338	7.33E-04	0.02561
	GO:0003677~DNA binding	17	2.8692458	9.81E-04	0.04059
	GO:0016563~transcription activator activity	8	7.6765705	0.0011010	0.05698
	GO:0016481~negative regulation of transcription	8	7.3681917	0.0025603	0.09544
5	GO:0010604~positive regulation of macromolecule metabolic process	10	4.9329054	0.0033599	0.13311
	GO:0010629~negative regulation of gene expression	8	6.7103174	0.0040925	0.17170
	GO:0045934~negative regulation of nucleobase, nucleoside, nucleotide and nucleic acid metabolic process	8	6.6054687	0.0042769	0.18940
	GO:0051172~negative regulation of nitrogen compound metabolic process	8	6.5163776	0.0044211	0.20609

Table S4 (C): Enriched GO terms of identified modules in dataset I

ID	Term	Count	Fold Enrichment	Benjamini	FDR
5	GO:0010558~negative regulation of macromolecule biosynthetic process	8	6.1828153	0.0058264	0.28527
	GO:0031327~negative regulation of cellular biosynthetic process	8	6.0285204	0.0064962	0.33324
	GO:0009890~negative regulation of biosynthetic process	8	5.9022687	0.0070736	0.37938
	GO:0003702~RNA polymerase II transcription factor activity	6	9.6743666	0.0054231	0.33705
	GO:0003779~actin binding	11	18.253195	7.14E-09	8.19E-08
	GO:0008092~cytoskeletal protein binding	11	11.806630	2.48E-07	5.69E-06
	GO:0005912~adherens junction	7	28.862580	7.77E-06	7.80E-05
	GO:0070161~anchoring junction	7	26.009883	7.21E-06	1.45E-04
	GO:0005924~cell-substrate adherens junction	6	36.175471	1.42E-05	4.29E-04
	GO:0030055~cell-substrate junction	6	34.2375	1.41E-05	5.65E-04
	GO:0015629~actin cytoskeleton	7	16.630855	4.00E-05	0.00201
	GO:0007010~cytoskeleton organization	8	10.792181	0.0029277	0.00588
	GO:0043232~intracellular non-membrane-bounded organelle	14	3.4466101	1.44E-04	0.00866
	GO:0043228~non-membrane-bounded organelle	14	3.4466101	1.44E-04	0.00866
	GO:0016323~basolateral plasma membrane	6	18.889655	1.51E-04	0.01059
	GO:0005925~focal adhesion	5	31.328431	1.91E-04	0.01534
	GO:0030016~myofibril	5	28.788288	2.37E-04	0.02143
	GO:0044449~contractile fiber part	5	28.278761	2.29E-04	0.02300
	GO:0030036~actin cytoskeleton organization	6	15.615236	0.0065640	0.03962
	GO:0043292~contractile fiber	5	26.409090	2.73E-04	0.03012
	GO:0030029~actin filament-based process	6	14.643333	0.0067001	0.05393
	GO:0010604~positive regulation of macromolecule metabolic process	9	6.1768555	0.0054772	0.05507
	GO:0005856~cytoskeleton	10	4.6278059	6.20E-04	0.07472
	GO:0030054~cell junction	7	8.6364864	6.46E-04	0.08437
	GO:0019900~kinase binding	5	15.110567	0.0080340	0.27725
6	GO:0008092~cytoskeletal protein binding	19	9.7887698	2.02E-11	1.39E-10
	GO:0003779~actin binding	16	12.744049	4.73E-11	6.48E-10

Table S4 (C): Enriched GO terms of identified modules in dataset I

ID	Term	Count	Fold Enrichment	Benjamini	FDR
6	GO:0044449~contractile fiber part	9	23.675241	4.22E-07	3.22E-06
	GO:0043292~contractile fiber	9	22.109936	3.64E-07	5.55E-06
	GO:0005924~cell-substrate adherens junction	8	22.434401	2.47E-06	5.65E-05
	GO:0030016~myofibril	8	21.423842	2.55E-06	7.79E-05
	GO:0030055~cell-substrate junction	8	21.232558	2.17E-06	8.29E-05
	GO:0005856~cytoskeleton	19	4.0896889	2.65E-06	1.21E-04
	GO:0007010~cytoskeleton organization	12	7.9219207	1.60E-04	2.61E-04
	GO:0015629~actin cytoskeleton	10	11.050402	3.88E-06	2.07E-04
	GO:0005912~adherens junction	8	15.342235	1.26E-05	7.66E-04
	GO:0005925~focal adhesion	7	20.399908	1.62E-05	0.001115 136
	GO:0070161~anchoring junction	8	13.825851	2.03E-05	0.001545 463
	GO:0016323~basolateral plasma membrane	8	11.714514	5.56E-05	0.004662 632
	GO:0007517~muscle organ development	8	10.912977	0.0031345	0.010214
	GO:0006936~muscle contraction	7	13.168683	0.0039353	0.019243
	GO:0003012~muscle system process	7	11.992907	0.0050215	0.032755
	GO:0034330~cell junction organization	5	25.248226	0.0078042	0.063712
	GO:0051270~regulation of cell motion	7	10.439422	0.0072990	0.071485
	GO:0005886~plasma membrane	26	2.0462407	6.68E-04	0.061088
	GO:0051271~negative regulation of cell motion	5	22.843633	0.0082799	0.09464
	GO:0010035~response to inorganic substance	7	9.8283341	0.0076594	0.10002
	GO:0044057~regulation of system process	8	7.4519038	0.0080802	0.11872
	GO:0043228~non-membrane-bounded organelle	20	2.2901064	0.0027969	0.27728
	GO:0043232~intracellular non-membrane-bounded organelle	20	2.2901064	0.0027969	0.27728
	GO:0030017~sarcomere	5	15.166112	0.0032775	0.34988
	GO:0044459~plasma membrane part	18	2.4287810	0.0033480	0.3828
7	GO:0003779~actin binding	15	13.892495	1.43E-10	1.03E-09

Table S4 (C): Enriched GO terms of identified modules in dataset I

ID	Term	Count	Fold Enrichment	Benjamini	FDR
7	GO:0008092~cytoskeletal protein binding	16	9.5850867	1.78E-09	2.55E-08
	GO:0044449~contractile fiber part	7	19.312324	2.18E-04	0.00153
	GO:0015629~actin cytoskeleton	9	10.430501	1.29E-04	0.00182
	GO:0043292~contractile fiber	7	18.035476	1.09E-04	0.00229
	GO:0044459~plasma membrane part	21	2.9718012	8.65E-05	0.00243
	GO:0006937~regulation of muscle contraction	6	25.051851	0.0029300	0.0053
	GO:0010035~response to inorganic substance	8	11.731598	0.0016872	0.00611
	GO:0007010~cytoskeleton organization	10	6.895005	0.0025599	0.01391
	GO:0034329~cell junction assembly	5	36.661246	0.0019304	0.01398
	GO:0006936~muscle contraction	7	13.753957	0.0016066	0.01454
	GO:0005886~plasma membrane	26	2.1460573	5.38E-04	0.01892
	GO:0003012~muscle system process	7	12.525925	0.0022827	0.02480
	GO:0005509~calcium ion binding	13	4.2710479	0.0012661	0.02727
	GO:0030016~myofibril	6	16.851680	6.75E-04	0.02853
	GO:0034330~cell junction organization	5	26.370370	0.0041383	0.0525
	GO:0005856~cytoskeleton	15	3.3861994	0.0010574	0.05211
	GO:0044057~regulation of system process	8	7.7830996	0.0053322	0.08701
	GO:0051924~regulation of calcium ion transport	5	21.784219	0.0061565	0.11166
	GO:0030036~actin cytoskeleton organization	7	9.3113077	0.0065518	0.13073
	GO:0006816~calcium ion transport	6	12.702347	0.0065963	0.14357
	GO:0030029~actin filament-based process	7	8.7317657	0.0078895	0.18611
	GO:0010959~regulation of metal ion transport	5	18.556927	0.0082182	0.20879
	GO:0048471~perinuclear region of cytoplasm	7	7.5774051	0.0054274	0.30598
	GO:0005924~cell-substrate adherens junction	5	14.705476	0.0062559	0.39677
	GO:0030055~cell-substrate junction	5	13.917682	0.0069428	0.48920
	GO:0016323~basolateral plasma membrane	6	9.2144659	0.0063514	0.49213

Table S4 (C): Enriched GO terms of identified modules in dataset I

ID	Term	Count	Fold Enrichment	Benjamini	FDR
9	GO:0003779~actin binding	16	12.022687	2.29E-10	1.69E-09
	GO:0008092~cytoskeletal protein binding	17	8.2626160	4.90E-09	7.25E-08
	GO:0006816~calcium ion transport	8	16.215762	3.84E-04	7.10E-04
	GO:0005856~cytoskeleton	18	3.7022447	2.10E-04	0.00161
	GO:0007010~cytoskeleton organization	11	7.2617606	6.55E-04	0.00242
	GO:0015674~di-, tri-valent inorganic cation transport	8	13.083172	5.43E-04	0.00301
	GO:0043228~non-membrane-bounded organelle	23	2.5165725	5.53E-04	0.01272
	GO:0043232~intracellular non-membrane-bounded organelle	23	2.5165725	5.53E-04	0.01272
	GO:0015629~actin cytoskeleton	8	8.4474184	0.0013057	0.04005
	GO:0030055~cell-substrate junction	6	15.216666	0.0012255	0.04699
	GO:0016323~basolateral plasma membrane	7	9.7946360	0.0016633	0.07654
	GO:0045121~membrane raft	6	11.917948	0.0027978	0.1502
	GO:0005912~adherens junction	6	10.995268	0.0035711	0.21916
	GO:0070161~anchoring junction	6	9.9085271	0.0051448	0.35525
	GO:0005886~plasma membrane	25	1.8800929	0.0070906	0.54403
	GO:0005924~cell-substrate adherens junction	5	13.398322	0.0066870	0.56419
	GO:0030016~myofibril	5	12.794794	0.0072964	0.67142
	GO:0044449~contractile fiber part	5	12.568338	0.0072053	0.71809
	GO:0043292~contractile fiber	5	11.737373	0.0080736	0.92784
10	GO:0008092~cytoskeletal protein binding	15	7.2905435	1.07E-06	8.10E-06
	GO:0003779~actin binding	12	9.0170158	4.05E-06	6.14E-05
	GO:0015629~actin cytoskeleton	10	11.050402	2.67E-05	2.07E-04
	GO:0005856~cytoskeleton	15	3.2287018	0.0060867	0.09456
	GO:0005912~adherens junction	6	11.506676	0.0074944	0.17470
	GO:0070161~anchoring junction	6	10.369388	0.0091294	0.28383
	GO:0044449~contractile fiber part	5	13.152912	0.0096490	0.59918
	GO:0016323~basolateral plasma membrane	6	8.7858861	0.0087032	0.60769
	GO:0043292~contractile fiber	5	12.283298	0.0099924	0.77509

Table S4 (D): Identified modules using negative PCC of miRNA-mRNA in dataset I

ID	microRNA	TF	nTF	Score	P value
1	hsa-let-7e* hsa-miR-181c* hsa-miR-196a	HAND1,JUND,RFX3,SOX9,SP3,SREBF2,TCF7	ANXA6,ATP2B4,CCND2,CHRDL1,CNN1,CXCL11,DPYSL3,EGR2,FLNA,JAZF1,LMOD1,MSRB3,MYLK,RBPMS,SLC8A1,SORBS1,SVIL,TAGLN,TNS1,TPM1,TSPAN18,VCL	0.8652	1.90E-03
2	hsa-miR-136 hsa-miR-198 hsa-miR-383	MEIS1,RFX3,SOX9	ACTA2,ATP2B4,CALD1,CAP2,CAV1,CCND2,CDC42EP3,CFL2,CHRDL1,CLU,CNN1,CORO1C,DDR2,DMD,DPYSL3,FHL1,FLNA,FRMD6,LMOD1,MAP1B,MSRB3,MYL9,MYLK,MYOCD,PALLD,PCP4,RBPMS,SEC23A,SLC8A1,SORBS1,SVIL,TIMP2,TLN1,TNS1,TPM1,VCL	0.896	1.00E-03
3	hsa-miR-181c* hsa-miR-193a-5p hsa-miR-383	FOSL1,HAND1,JUND,NFE2L1,NFKB2,RUNX3,STAT3	ACTN1,AHNAK2,ANXA6,BNC2,CHRDL1,CLU,COL4A2,COL6A1,DES,DKK3,EDNRA,FLNA,FLNC,FZD7,GSN,HEPH,HTRA1,ITGA9,ITIH5,JAZF1,KCNMB1,LGALS1,LMOD1,MAP1B,MCAM,MFGE8,MRVI1,MYL9,MYLK,NID1,PMP22,RBPMS,SLC24A3,SLC8A1,TIMP2,TNS1,TPM2,TSPAN18,WFDC1	0.865	1.10E-03
4	hsa-let-7e* hsa-miR-885-5p	ELF4,HAND1,HNF4A,NFATC2,NFE2L3,RFX3,SOX9	ASCC1,ATP6V1E1,CLASP1,CLCA2,DCTN2,DLST,DYNC1H1,FXYD6,GPX2,KIAA0430,MYST2,PARD3,PCDH10,PPP3CB,RBM23,SDHD,SERPINA11,SIGLEC12,TMEM132A,TOR1AIP1,ZFHX4	0.8255	1.00E-03

Table S4 (D): Identified modules in dataset I

ID	microRNA	TF	nTF	Score	P value
5	hsa-miR-101* hsa-miR-136*	RFX3,SOX9	ACTN1,ANXA6,ATP2B4,CAV1,CCND2,CNN1,CSRP1,FLNA,GSN,ILK,SORBS1,SPTAN1,SVIL,TAGLN,TLN1,TNS1,TPM2	0.8844	3.60E-03
6	hsa-miR-101* hsa-miR-196a hsa-miR-198	HOXA7,MEIS1,RFX3,SOX9,ZEB1	ACTG2,ACTN1,ANXA6,ATP2B4,CALD1,CAMK2G,CAV1,CCND2,CHRDL1,CLU,CNN1,CORO1C,CRYAB,CSRP1,DDR2,DES,DPYSL3,DUSP3,EDNRA,EYA4,FHL1,FLNA,FLNC,ILK,KCNMB1,LMOD1,MAOB,MATN2,MEIS2,MSRB3,MYLK,NEXN,NPTN,PALLD,PARVA,PPP1R3C,PRNP,RBPMS,SLC8A1,SVIL,TAGLN,TGFB2,TGFBR3,TIMP2,TLN1,TNS1,TPM1,TSPAN2,VCL	0.8714	1.40E-03
7	hsa-miR-198 hsa-miR-335*	SOX9	ACTN1,ADRA1A,ANXA6,ATP2B4,BNC2,CAV1,CHRDL1,CLU,CNN1,CNTN1,CORO1C,DDR2,DPYSL3,EDNRA,FAT4,FBN1,FLNA,GNAL,GSN,ITGB3,ITIH5,KCNMA1,LAMA4,LMOD1,MAP1B,MRVI1,MSRB3,MYH11,MYLK,MYOCD,PDZRN4,PMP22,RBMS2,RGN,SLC8A1,SORBS1,SSPN,SVIL,TIMP2,TMEM47,TNS1,TPM1,TPM2,TRPC1,TRPC4,VCL	0.8924	4.00E-04

Table S4 (D): Identified modules in dataset I

ID	microRNA	TF	nTF	Score	P value
8	hsa-miR-136 hsa-miR-193a-5p hsa-miR-214*	EGR3,FOSL1,HAND1,IRF4,POU4F1,SOX9,TGIF1	ADCY5,AHNAK2,CAV1,CHRDL1,CLU,CNN1,COL4A2,COL6A1,COL6A2,DBNDD2,DES,DKK3,DNAJB5,EFEMP2,ENO2,FGFR1,FHOD3,FLNC,FZD7,GAS6,GPR124,GSN,HEPH,ITGA7,ITGA9,ITGB3,ITIH5,JAZF1,LMOD1,MARVELD1,MCAM,MKX,MRGPRF,MRVI1,NFASC,PNMA1,PRICKLE2,RARRES2,SLC24A3,ST5,TSPAN18,WFDC1	0.8576	5.00E-04
9	hsa-miR-155 hsa-miR-27b*		ABCC9,ANXA6,ATP2B4,C20orf194,CACHD1,CALD1,CAMK2G,CAP2,CAV1,CCND2,CFL2,CLIP4,CORO1C,DDR2,DMD,DPYSL3,EDNRA,EPB41L2,FRMD6,KLHL5,LTBP1,MAP1B,MFN2,MSRB3,NT5E,PALLD,PDGFC,PLS3,PRNP,PSIP1,RBMS2,RBMS3,RBPMS,SEC23A,SLC8A1,SORBS1,SPARCL1,SPTAN1,SVIL,TEAD1,TIMP2,TLN1,TNS1,TRPC1,TRPC4,TSPAN2,VCL,WWTR1,ZNF483	0.8871	5.00E-04

Table S4 (D): Identified modules in dataset I

ID	microRNA	TF	nTF	Score	P value
10	hsa-let-7f-1* hsa-miR-133b hsa-miR-625	ESR1,IRF1,MAFF,NR5A2,RFX3,TFDP1,ZEB1	ACSL4,C20orf194,CAP2,CDC42EP3,CDS2,CFL2,CLIC4,CLIP4,CNTN1,CORO1C,CRYAB,CSRP1,DPYSL3,DUSP3,EPB41L2,FAM92A1,FCGBP,HECTD2,ITSN1,JAK1,KPNA3,LPP,MBNL2,MEIS2,NEXN,NPTN,PALLD,PBX1,PCDH18,PDGFC,PIK3R1,PLS3,PLSCR4,PPP1R12A,PRNP,PRRT2,PSIP1,PTPLA,RBM9,RBMS3,RNF180,RSU1,SEC23A,SLC8A1,SORBS1,SPTAN1,TEAD1,TLN1,TPM1,TRPC1,VCL,WWTR1,ZNF483	0.843	9.00E-04

Table S5. Summary of regulatory interactions in Dataset II

Module ID	# Nodes[a)]	#Interactions[b)]	#PCC & Binding[c)]	#PCC[d)]	# Binding[e)]
1	2/6/15	111	27	84	0
2	2/6/08	34	21	13	0
3	2/5/33	559	86	473	0
4	2/6/21	286	72	214	0
5	2/6/10	107	54	53	0
6	2/6/13	154	71	83	0
7	2/6/10	96	48	48	0
8	2/6/17	180	67	113	0
9	2/6/15	160	47	113	0
10	2/6/13	138	57	81	0

a) The numbers of miRNAs, TF-genes, nTF-genes
b) The number of interactions
c) The number of interactions with support of both significant PCC and predicted binding
d) The number of interactions with support of only significant PCC
e) The number of interactions with support of only predicted binding

Table S6 (A): Table of identified modules in dataset II

ID	microRNA	TF	nTF	Score	P value
1	hsa-miR-15a hsa-miR-19a	EBF1,EGR3,FOXA1,NFE2L3,TFAP2C,ZBTB7A	ADD3,ATP5A1,DNAJC3,FABP1,ITM2B,KLHDC2,NDUFS4,PAN3,PCBP1,PCYOX1,PERP,SACM1L,SLC26A3,SRP9,TSPYL1	0.8364	4.80E-03
2	hsa-miR-19a hsa-miR-19b	IRF7,IRF8,MYCN,TFAP2C,TP53,ZBTB7A	EFHA1,PAN3,SACM1L,SEMG1,SEMG2,SLC12A3,SLC34A1,TSPYL1	0.7938	1.50E-02
3	hsa-miR-136 hsa-miR-15a	ETS2,HMGA1,MYB,NFE2L3,TFAP2C	ADD3,ATP5A1,C16orf61,C8orf59,CHMP5,COL5A2,COMMD3,COX7A2L,DNAJC3,EFHA1,GBAS,ITM2B,KIAA1430,MTCH1,NDUFA4,NDUFC1,NDUFS4,PAN3,PCBP1,PCYOX1,RAD51L1,SACM1L,SEPT2,SLC25A3,SRP9,SUV420H1,TMEM123,TMEM14C,TSC22D1,TSPYL1,USP33,VPS11,ZC3H15	0.8233	3.10E-03
4	hsa-miR-107 hsa-miR-15a	ARNT2,HMGA1,MYB,NFE2L3,TFAP2C,ZBTB7A	C20orf30,CD164,DDX5,EFHA1,EIF4A2,FOXJ3,GPR17,KIAA1033,KLHDC2,MORF4L1,MTCH1,NPTN,PCBP1,PCNP,PERP,SLC25A3,SRP9,TMEM14C,TSPYL1,UBE4A,ZFAND3	0.8088	6.90E-03
5	hsa-miR-128a hsa-miR-132	FOXA1,HMGA1,MYB,NHLH1,RFXAP,SMAD3	CDKN1B,FBXL3,FUBP1,GBAS,KIAA1430,PAN3,PCYOX1,SACM1L,TSPYL1,USP33	0.8177	7.20E-03
6	hsa-miR-19a hsa-miR-19b	ARNT2,GATA3,MYB,MYC,RFXAP,TCF4	ANXA7,C20orf30,DDX5,EIF4A2,EIF4G2,FOXJ3,ITM2B,MORF4L1,NPTN,PCNP,PCYOX1,SEPT10,TJP1	0.8406	6.00E-03

Table S6 (A): Table of identified modules in dataset II

ID	microRNA	TF	nTF	Score	P value
7	hsa-miR-1 hsa-miR-141	ARNT2,HMGA1,MYC, TFAP2C,TP53,ZBTB7A	C20orf30,DDX5,FBXW11,FOXJ3,KIAA0776,MORF4L1,NPTN,PCNP,PCYOX1,PERP	0.8117	7.40E-03
8	hsa-miR-15a hsa-miR-206	EBF1,HMGA1,NHLH1, RFXAP,SMAD3,TFAP2C	ADIPOR2,COL1A1,COL5A2, DDX5,DSTN,EIF4A2,FBXW11,GOLPH3,HIAT1,NPTN,PCBP1,PCMTD1,PCNP,PCYOX1,SEPT10,VEZF1,ZNF83	0.8174	4.00E-03
9	hsa-miR-141 hsa-miR-200a	ATF6,EBF1,FOXA1,MYCN,TFAP2C,ZBTB7A	ADD3,C22orf32,FUBP1,GBAS,HOXD11,KLHDC2,MKNK2,PAN3,PFKFB4,SACM1L,SUV420H1,TMBIM1,TMEM123,TSPYL1,ZC3H15	0.8164	4.80E-03
10	hsa-miR-19a hsa-miR-19b	ARNT2,CEBPB,HMGA1,TFAP2C,TP53,ZBTB7A	ADD3,C16orf61,CHMP5,FOXJ3,GBAS,GPR17,KIAA1430,KLHDC2,NDUFS4,PAN3,PERP,SACM1L,USP33	0.817	6.20E-03

Table S6 (B): GO term & KEGG pathway enrichment of identified modules in dataset II

ID	Term and Pathway	Count	Fold Enrichment	P Value
1	GO:0003700~transcription factor activity	6	4.44	0.006825
	GO:0030528~transcription regulator activity	7	3.34	0.009808
2	GO:0006357~regulation of transcription from RNA polymerase II promoter	6	8.59	0.000255
	GO:0000122~negative regulation of transcription from RNA polymerase II promoter	4	15.65	0.001450
	GO:0045892~negative regulation of transcription, DNA-dependent	4	11.69	0.003331
	GO:0051253~negative regulation of RNA metabolic process	4	11.50	0.003493
	GO:0016481~negative regulation of transcription	4	9.07	0.006792
	GO:0030528~transcription regulator activity	6	3.96	0.008331
	GO:0010629~negative regulation of gene expression	4	8.26	0.008795
	GO:0045934~negative regulation of nucleobase, nucleoside, nucleotide and nucleic acid metabolic process	4	8.13	0.009184
	GO:0051172~negative regulation of nitrogen compound metabolic process	4	8.02	0.009533
	GO:0003700~transcription factor activity	5	5.12	0.009600
3	GO:0005743~mitochondrial inner membrane	7	11.25	0.000022
	GO:0019866~organelle inner membrane	7	10.46	0.000033
	GO:0031966~mitochondrial membrane	7	8.73	0.000089
	GO:0044455~mitochondrial membrane part	5	19.66	0.000094
	hsa05012:Parkinson's disease	5	16.55	0.000110
	hsa00190:Oxidative phosphorylation	5	16.30	0.000117
	GO:0005740~mitochondrial envelope	7	8.21	0.000125
	GO:0031090~organelle membrane	10	4.49	0.000140
	GO:0005746~mitochondrial respiratory chain	4	30.73	0.000255
	hsa05010:Alzheimer's disease	5	13.00	0.000282
	GO:0070469~respiratory chain	4	26.22	0.000407
	hsa05016:Huntington's disease	5	11.77	0.000412
	GO:0005739~mitochondrion	9	4.07	0.000775
	GO:0044429~mitochondrial part	7	5.78	0.000822
	GO:0031967~organelle envelope	7	5.55	0.001020
	GO:0031975~envelope	7	5.53	0.001037
	GO:0006119~oxidative phosphorylation	4	19.04	0.001059
3	GO:0006091~generation of precursor metabolites and energy	5	7.45	0.003713
4	GO:0030528~transcription regulator activity	8	3.82	0.001932
5	GO:0030528~transcription regulator activity	7	4.62	0.001223
	GO:0016563~transcription activator activity	4	9.74	0.005561
	GO:0003700~transcription factor activity	5	5.12	0.009600

Table S6 (B): GO term & KEGG pathway enrichment of identified modules in dataset II

ID	Term and Pathway	Count	Fold Enrichment	P Value
6	GO:0030528~transcription regulator activity	8	4.04	0.001263
	GO:0008134~transcription factor binding	5	7.44	0.003001
	GO:0003700~transcription factor activity	6	4.70	0.005130
7	GO:0030528~transcription regulator activity	8	5.72	0.000062
	GO:0044451~nucleoplasm part	5	10.47	0.000599
	GO:0003700~transcription factor activity	6	6.66	0.000745
	GO:0045449~regulation of transcription	9	3.34	0.000941
	GO:0006350~transcription	8	3.68	0.001557
	GO:0045941~positive regulation of transcription	5	8.57	0.001582
	GO:0010628~positive regulation of gene expression	5	8.32	0.001765
	GO:0045935~positive regulation of nucleobase, nucleoside, nucleotide and nucleic acid metabolic process	5	7.74	0.002296
	GO:0051173~positive regulation of nitrogen compound metabolic process	5	7.50	0.002577
	GO:0010557~positive regulation of macromolecule biosynthetic process	5	7.39	0.002727
	GO:0031981~nuclear lumen	6	4.81	0.002877
	GO:0031328~positive regulation of cellular biosynthetic process	5	7.05	0.003228
	GO:0005654~nucleoplasm	5	6.59	0.003377
	GO:0009891~positive regulation of biosynthetic process	5	6.95	0.003402
	GO:0006355~regulation of transcription, DNA-dependent	7	3.82	0.003785
	GO:0006357~regulation of transcription from RNA polymerase II promoter	5	6.65	0.004005
	GO:0051252~regulation of RNA metabolic process	7	3.73	0.004244
	GO:0003677~DNA binding	7	3.25	0.006735
	GO:0070013~intracellular organelle lumen	6	3.92	0.007117
	GO:0010604~positive regulation of macromolecule metabolic process	5	5.64	0.007210
	GO:0043233~organelle lumen	6	3.83	0.007859
	GO:0008134~transcription factor binding	4	8.44	0.007981
	GO:0031974~membrane-enclosed lumen	6	3.76	0.008556
	GO:0045893~positive regulation of transcription, DNA-dependent	4	8.10	0.009568
	GO:0051254~positive regulation of RNA metabolic process	4	8.04	0.009790

Table S6 (B): GO term & KEGG pathway enrichment of identified modules in dataset II

ID	Term and Pathway	Count	Fold Enrichment	P Value
7	hsa04310:Wnt signaling pathway	3	16.84	0.008259
	hsa05200:Pathways in cancer	3	7.75	0.036417
8	GO:0030528~transcription regulator activity	9	3.68	0.001150
	GO:0032535~regulation of cellular component size	4	9.51	0.007042
9	GO:0003700~transcription factor activity	7	6.21	0.000314
	GO:0030528~transcription regulator activity	8	4.58	0.000468
	GO:0045449~regulation of transcription	9	2.93	0.003257
	GO:0006350~transcription	8	3.22	0.004387
	GO:0003677~DNA binding	8	2.97	0.006288
	GO:0006357~regulation of transcription from RNA polymerase II promoter	5	5.81	0.007016
	GO:0006355~regulation of transcription, DNA-dependent	7	3.34	0.008751
	GO:0051252~regulation of RNA metabolic process	7	3.26	0.009759
10	GO:0003700~transcription factor activity	6	6.15	0.001198
	GO:0030528~transcription regulator activity	7	4.62	0.001223
	GO:0006355~regulation of transcription, DNA-dependent	7	3.82	0.003785
	GO:0006357~regulation of transcription from RNA polymerase II promoter	5	6.65	0.004005
	GO:0051252~regulation of RNA metabolic process	7	3.73	0.004244
	GO:0006350~transcription	7	3.22	0.008922
	GO:0045893~positive regulation of transcription, DNA-dependent	4	8.10	0.009568

Supplementary Figures

Figure S1: Score track of local search for 10 modules

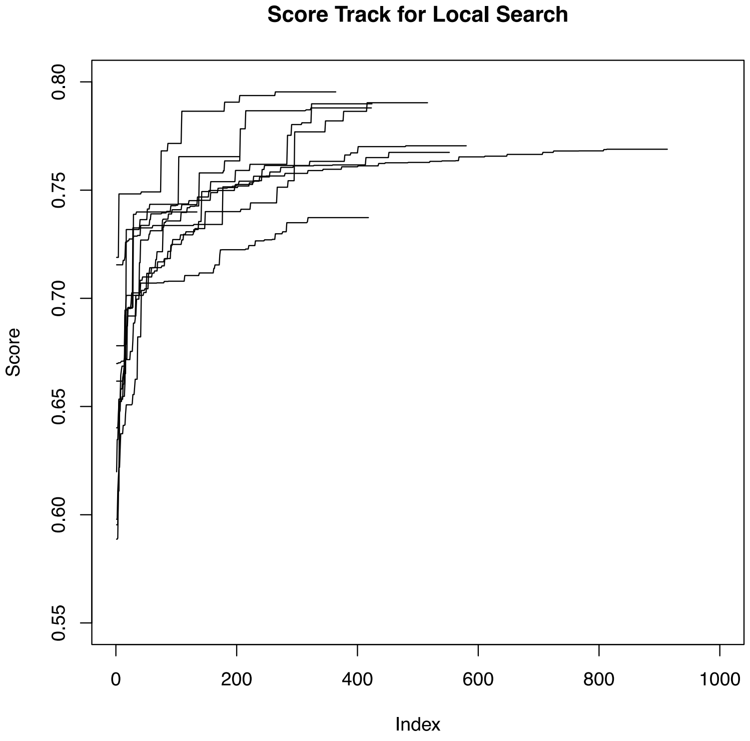

Figure S2: Visualization of identified modules in dataset I: (A) Module 2; (B) Module 3; C) Module 4; (D) Module 5

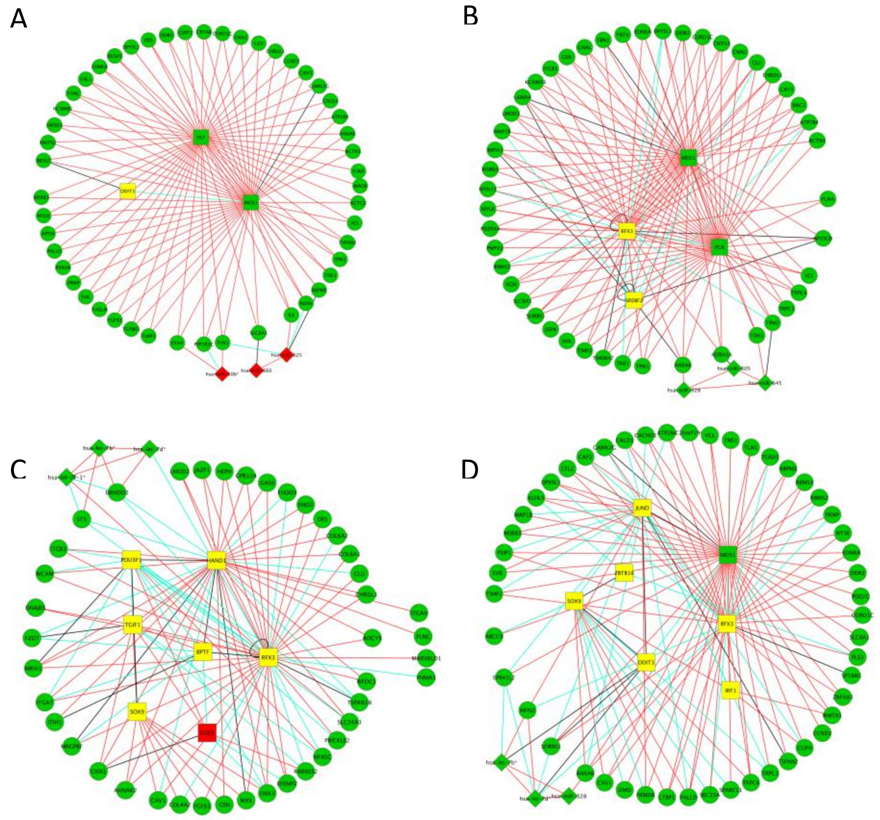

Figure S3: Visualization of identified modules in dataset I: (A) Module 6; (B) Module 7; (C) Module 8; (D) Module 9

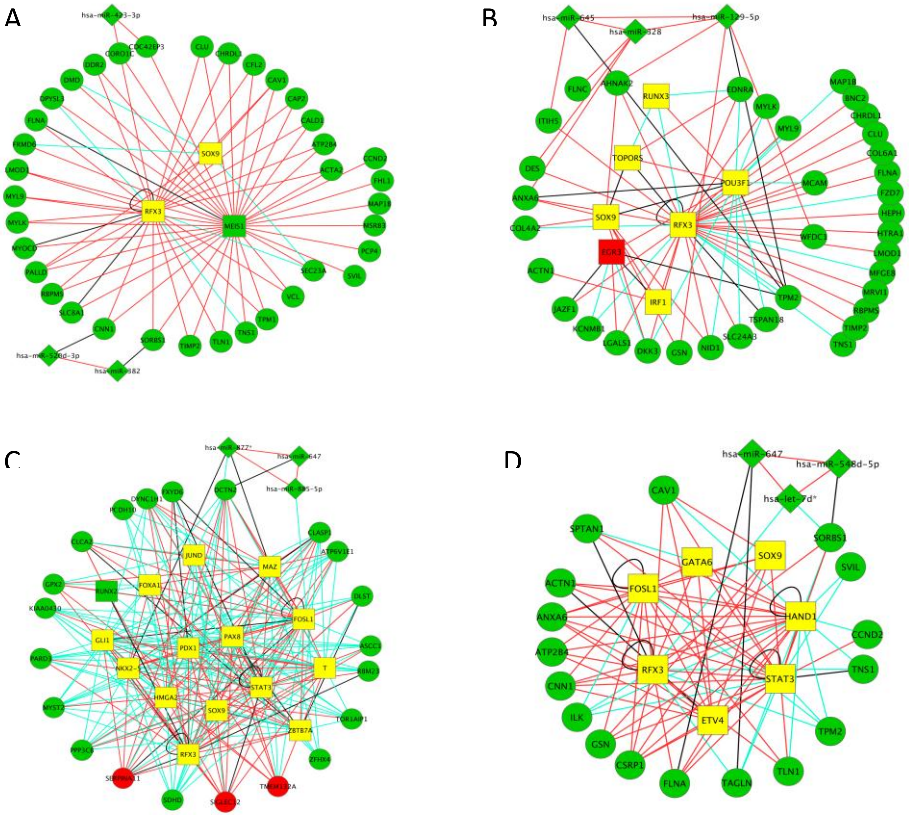

Figure S4: Visualization of modules in dataset II: (A) Module 1; (B) Module 2; (C) Module 3; (D) Module 4; (E) Module 5; (F) Module 6

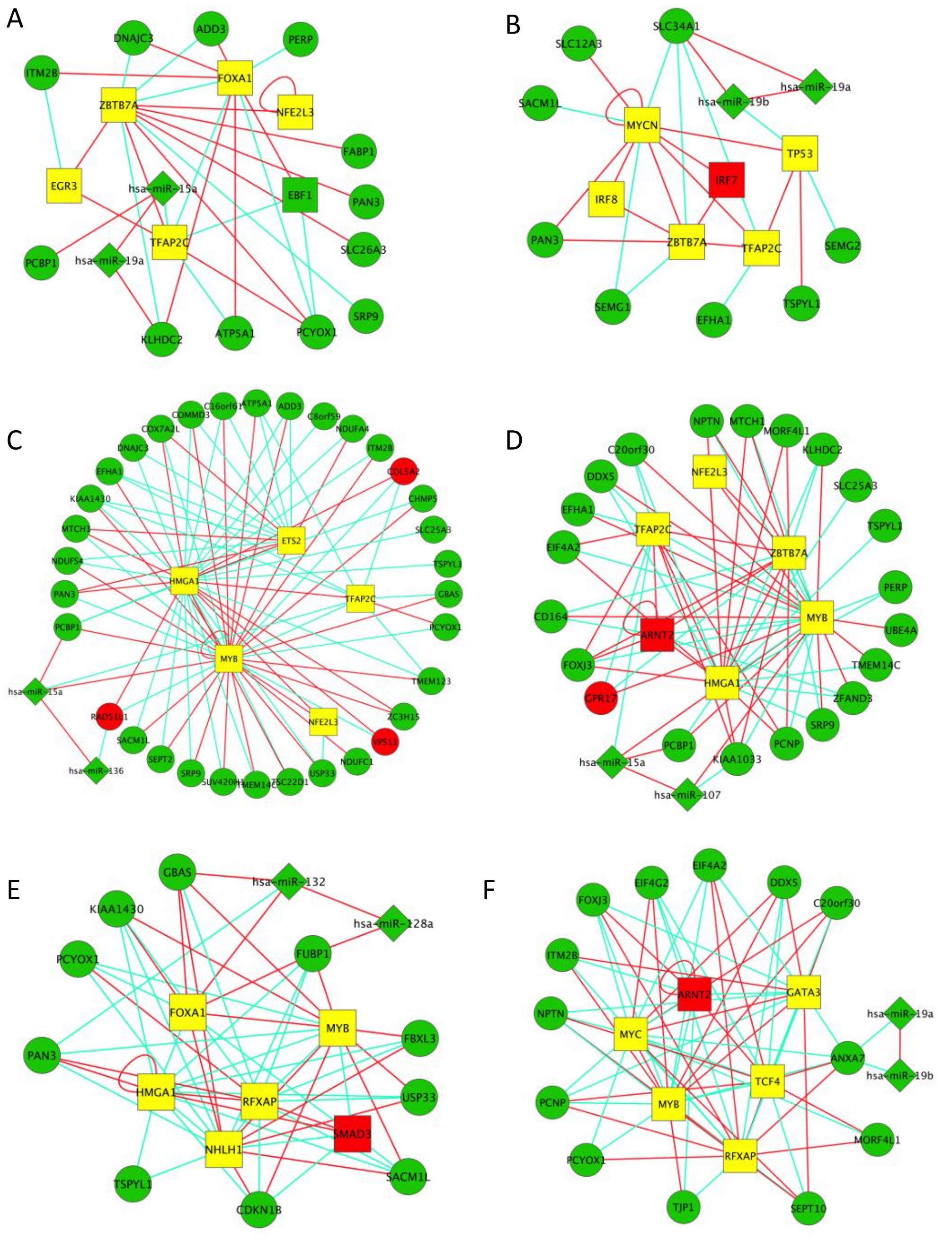

Figure S5. Visualization of modules in Dataset II: (A) Module 7; (B) Module 8; (C) Module 9; (D) Module 10

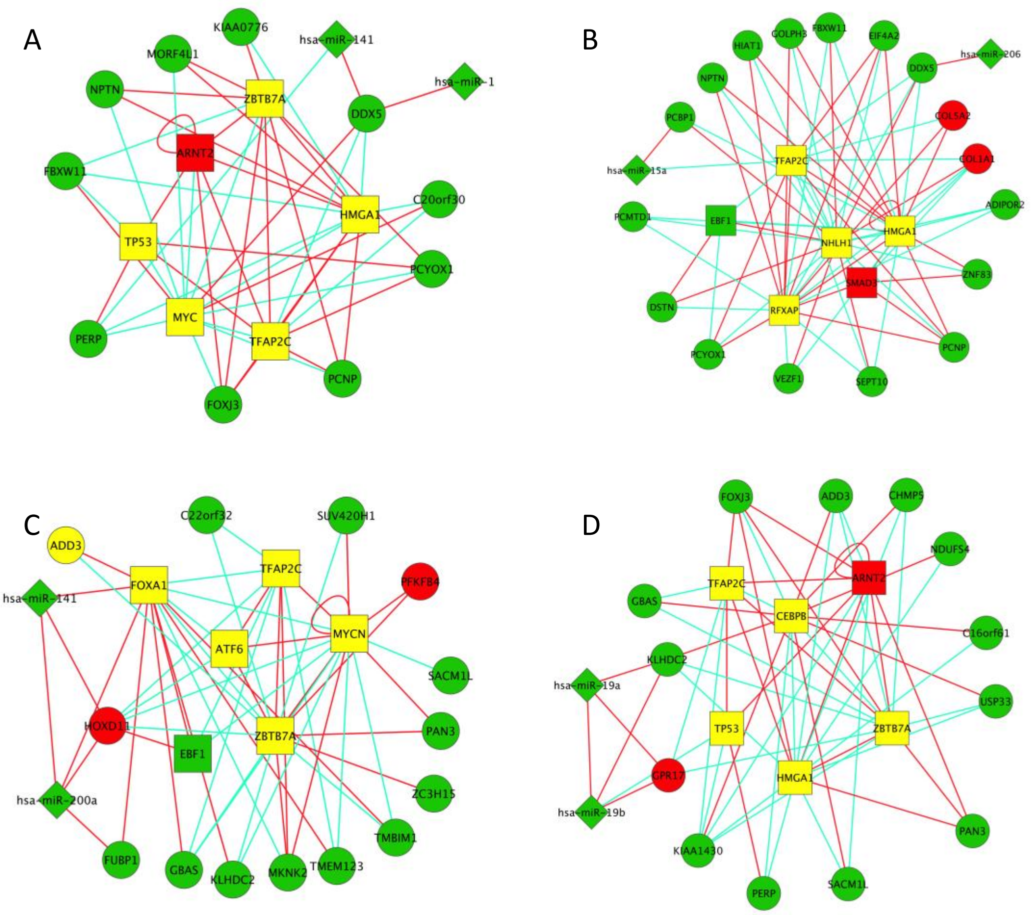

References

1. Bandi N, Zbinden S, Gugger M, Arnold M, Kocher V, Hasan L, Kappeler A, Brunner T, Vassella E: **miR-15a and miR-16 Are Implicated in Cell Cycle Regulation in a Rb-Dependent Manner and Are Frequently Deleted or Down-regulated in Non–Small Cell Lung Cancer**. *Cancer Research* 2009, **69**(13):5553-5559.
2. Cho J-H, Gelinas R, Wang K, Etheridge A, Piper M, Batte K, Dakhlallah D, Price J, Bornman D, Zhang S *et al*: **Systems biology of interstitial lung diseases: integration of mRNA and microRNA expression changes**. *BMC Medical Genomics* 2011, **4**(1):8.
3. Bonci D, Coppola V, Musumeci M, Addario A, Giuffrida R, Memeo L, D'Urso L, Pagliuca A, Biffoni M, Labbaye C *et al*: **The miR-15a-miR-16-1 cluster controls prostate cancer by targeting multiple oncogenic activities**. *Nature Medicine* 2008, **14**(11):1271-1277.
4. Polytarchou C, Iliopoulos D, Struhl K: **An integrated transcriptional regulatory circuit that reinforces the breast cancer stem cell state**. *Proceedings of the National Academy of Sciences* 2012, **109**(36):14470-14475.
5. Bhattacharya R, Nicoloso M, Arvizo R, Wang E, Cortez A, Rossi S, Calin GA, Mukherjee P: **MiR-15a and MiR-16 Control Bmi-1 Expression in Ovarian Cancer**. *Cancer Research* 2009, **69**(23):9090-9095.
6. Bandrés E, Cubedo E, Agirre X, Malumbres R, Zàrate R, Ramirez N, Abajo A, Navarro A, Moreno I, Monzó M *et al*: **Identification by Real-time PCR of 13 mature microRNAs differentially expressed in colorectal cancer and non-tumoral tissues**. *Molecular Cancer* 2006, **5**:29-10.
7. Hayashita Y, Osada H, Tatematsu Y, Yamada H, Yanagisawa K, Tomida S, Yatabe Y, Kawahara K, Sekido Y, Takahashi T: **A Polycistronic MicroRNA Cluster, miR-17-92, Is Overexpressed in Human Lung Cancers and Enhances Cell Proliferation**. *Cancer Research* 2005, **65**(21):9628-9632.
8. Pichiorri F, Suh S-S, Ladetto M, Kuehl M, Palumbo T, Drandi D, Taccioli C, Zanesi N, Alder H, Hagan JP *et al*: **MicroRNAs regulate critical genes associated with multiple myeloma pathogenesis**. *Proceedings of the National Academy of Sciences* 2008, **105**(35):12885-12890.
9. Abdueva D, Wing M, Schaub B, Triche T, Davicioni E: **Quantitative Expression Profiling in Formalin-Fixed Paraffin-Embedded Samples by Affymetrix Microarrays**. *The Journal of Molecular Diagnostics* 2010, **12**(4):409-417.
10. Bonnet S, Archer SL, Allalunis-Turner J, Haromy A, Beaulieu C, Thompson R, Lee CT, Lopaschuk GD, Puttagunta L, Bonnet S *et al*: **A Mitochondria-K+ Channel Axis Is Suppressed in Cancer and Its Normalization Promotes Apoptosis and Inhibits Cancer Growth**. *Cancer cell* 2007, **11**(1):37-51.
11. Lu S., Lee J., Revelo M., Wang X., Lu S., Z. D: **Smad3 is overexpressed in advanced human prostate cancer and necessary for progressive growth of prostate cancer cells in nude mice**. *Clin Cancer Res* 2007, **13**(19):5692-5702.
12. Oh I.H., E.P. R: **The myb gene family in cell growth, differentiation and apoptosis.** *Oncogene* 1999, **18**(19):3017-3033.
13. H. C: **Genetic regulation of cancer**. *Nature Education* 2008, **1**(1).
